# Association Between Adverse Early Life Factors and Telomere Length in Middle and Late Life

**DOI:** 10.1093/geroni/igae070

**Published:** 2024-08-10

**Authors:** Fengyu Lin, Jiefeng Luo, Yiqun Zhu, Huaying Liang, Dianwu Li, Duoduo Han, Qinyu Chang, Pinhua Pan, Yan Zhang

**Affiliations:** Center of Respiratory Medicine, Xiangya Hospital, Central South University, Changsha, Hunan, China; National Key Clinical Specialty, Branch of National Clinical Research Center for Respiratory Disease, Xiangya Hospital, Central South University, Changsha, Hunan, China; Department of Gynecology and Obstetrics, Xiangya Hospital, Central South University, Changsha, Hunan, China; International Collaborative Research Center for Medical Metabolomics, Xiangya Hospital, Central South University, Changsha, Hunan, China; Center of Respiratory Medicine, Xiangya Hospital, Central South University, Changsha, Hunan, China; Hunan Engineering Research Center for Intelligent Diagnosis and Treatment of Respiratory Disease, Changsha, Hunan, China; Center of Respiratory Medicine, Xiangya Hospital, Central South University, Changsha, Hunan, China; National Clinical Research Center for Geriatric Disorders, Xiangya Hospital, Central South University, Changsha, Hunan, China; Center of Respiratory Medicine, Xiangya Hospital, Central South University, Changsha, Hunan, China; National Key Clinical Specialty, Branch of National Clinical Research Center for Respiratory Disease, Xiangya Hospital, Central South University, Changsha, Hunan, China; Center of Respiratory Medicine, Xiangya Hospital, Central South University, Changsha, Hunan, China; National Key Clinical Specialty, Branch of National Clinical Research Center for Respiratory Disease, Xiangya Hospital, Central South University, Changsha, Hunan, China; Center of Respiratory Medicine, Xiangya Hospital, Central South University, Changsha, Hunan, China; National Key Clinical Specialty, Branch of National Clinical Research Center for Respiratory Disease, Xiangya Hospital, Central South University, Changsha, Hunan, China; Center of Respiratory Medicine, Xiangya Hospital, Central South University, Changsha, Hunan, China; FuRong Laboratory, Central South University, Changsha, Hunan, China; Center of Respiratory Medicine, Xiangya Hospital, Central South University, Changsha, Hunan, China; National Key Clinical Specialty, Branch of National Clinical Research Center for Respiratory Disease, Xiangya Hospital, Central South University, Changsha, Hunan, China

**Keywords:** Biological aging, Birth weight, Breastfeeding, Life expectancy, Maternal smoking

## Abstract

**Background and Objectives:**

Telomere length (TL) has been acknowledged as biomarker of biological aging. Numerous investigations have examined associations between individual early life factors and leukocyte TL; however, the findings were far from consistent.

**Research Design and Methods:**

We evaluated the relationship between individual and combined early life factors and leukocytes TL in middle and late life using data from the UK Biobank. The early life factors (eg, maternal smoking, breastfeeding, birth weight, and comparative body size and height to peers at age 10) were measured. The regression coefficients (β) and 95% confidence interval (CI) were applied to assess the link of the early life factors and TL in adulthood. Flexible parametric survival models incorporated age to calculate the relationship between early life factors and life expectancy.

**Results:**

Exposure to maternal smoking, lack of breastfeeding, low birth weight, and shorter height compared to peers at age 10 were identified to be associated with shorter TL in middle and older age according to the large population-based study with 197 504 participants. Individuals who experienced more than 3 adverse early life factors had the shortest TL in middle and late life (β = −0.053; 95% CI = −0.069 to −0.038; *p* < .0001), as well as an average of 0.54 years of life loss at the age of 45 and 0.49 years of life loss at the age of 60, compared to those who were not exposed to any early life risk factors.

**Discussion and Implications:**

Early life factors including maternal smoking, non-breastfed, low birth weight, and shorter height compared to peers at age 10 were associated with shorter TL in later life. In addition, an increased number of the aforementioned factors was associated with a greater likelihood of shorter TL in adulthood, as well as a reduced life expectancy.

**Translational Significance:** We comprehensively identified adverse early life factors of maternal smoking, non-breastfed, low birth weight, and shorter height compared to peers at age 10 were inversely correlated with shorter telomere length (TL) in middle and late life based on a large population from the UK Biobank. The study further provided evidence that an increased number of the aforementioned factors was associated with a greater likelihood of shorter TL in adulthood, as well as a reduced life expectancy. These findings highlight the significance of intervention in adverse early life factors to avoid premature biological aging and prolong life expectancy.

## Background

Given the increasing number of aging populations globally caused substantially medical and socioeconomic burden, there is a collective pursuit to attain a prolonged period of robust health while diminishing the susceptibility to age-related ailments. Telomere length (TL) is a biological aging marker that has been widely recognized and associated with a range of aging-related conditions (1–3). Cell senescence, cell cycle arrest, or cell apoptosis may ensue when a specific minimum length of telomeres is attained (4,5). Previous studies have established a correlation between a reduced TL and an increased risk of all-cause mortality (6), as well as multiple aging-related diseases such as certain cancers (7), Alzheimer’s disease (8), chronic lung (9) and heart disease (10), etc. TL can be affected by multiple factors, such as environmental conditions (11,12), infection (13–15), genetic predisposition (16,17), and stress (18), etc. Thus, the investigation of factors linked to TL may potentially facilitate the detection, intervention, and prevention of biological aging and age-related diseases.

An expanding corpus of evidences suggest that experiences in the initial decade of life are crucial for long-term health maintenance (19) and may have enduring impacts throughout an individual’s lifespan (20). Studies have indicated that certain early life factors, such as maternal smoking (16,21,22), breastfeeding (23,24), birth weight (25), and childhood growth (26,27) are highly associated with newborn and children’s TL; nevertheless, there is a dearth of sufficient evidence on the association between the aforementioned early life factors and TL in adulthood, particularly in middle and late life. A limited number of studies tried to examine the correlation between early life factors and TL in middle and late life; however, these studies raised findings far from consistent. For example, a newly published study from UK Biobank found early life tobacco exposure, including utero tobacco exposure and the age of smoking initiation in childhood had the highest accelerated biological aging that was partially predicted by TL (28). Although several studies identified breastfeeding was associated with longer telomeres in fetal and early childhood (23,24), relationship between breastfeeding and TL in adults was not observed in a birth cohort study with a relatively sample size of 1 759 individuals in Metropolitan Cebu, Philippines (15). Another study involving 1 562 Filipino individuals indicated that a greater birth weight could potentially serve as a predictor of longer TL in adults, while this association weakened and even disappeared after accounting for sufficient relevant confounding factors (29). A correlation between height at age 9 and TL at age 49 to 51 years was ever observed in women but not in men from an observational study with very small sample size (30); nevertheless, neither this study (30) nor others (31) discovered an association between birth weight and subsequent TL in later life. The discrepant findings could be partially attributed to the heterogeneity of populations’ characteristics and limited sample sizes. In addition, the majority of previous studies have concentrated on the relationship between individual early life factor, but not combined effect, and TL both in early and late life. Given that TL is a promising marker of biological aging that has been linked to subsequent mortality and chronic diseases, exploratory assessment is also necessary to determine the relationship between early life factors and life expectancy.

Herein, we aimed to illustrate the relationship between individual or combined early life factors including maternal smoking, breastfeeding, birth weight, and body size and height at age 10 and TL in middle and late life, as well as the life expectancy, using the large population-based data from UK Biobank.

## Method

### Study Design and Participants

This large population-based study was based on the data from the UK Biobank (under application no. 90060), which recruited over 500 000 individuals aged 37 to 73 at 22 medical centers throughout England, Wales, and Scotland from 2006 to 2010. The demographic variables including responses to questionnaires about demographics, lifestyle, and health-related factors, biological samples, and physical measurements, were collected. The UK Biobank had previously obtained ethical approval from the research ethics committee (reference 13/NW/0382). All participants signed the informed consents.

### Telomere Length

Comprehensive information regarding the measurement of TL has been previously documented (32). In brief, DNA was isolated from peripheral blood leukocytes, followed by quantitative polymerase chain reaction to evaluate leukocyte TL as a relative ratio of telomere repeat copy number (T) to a single-copy gene (S). In addition to being adjusted for technical variation, the gathered data were log transformed and *Z*-standardized. The *Z*-standardized log-TL measure (Data-Field 22192) was utilized in accordance with prior research (33–35).

### Early Life Factors

Five early life variables, including maternal smoking, breastfeeding during infancy, birth weight, and comparative body size and height at the age of 10, were assessed via questionnaire at the recruitment. Respondents were deemed to have a positive maternal smoking status if they answered “yes” to the question “Did your mother smoke regularly around the time when you were born?” (Data-Field 1787). Individuals who provided affirmative response to the question “Were you breastfed when you were a baby?” were classified as having received breastfed (Data-Field 1677).

The birth weight data were self-reported (Data-Field 20022) and dichotomized into 2 categories based on a well-known cutoff of 2 500 g, and a low birthweight was defined as an infant whose initial mass was less than 2 500 g (36). In response to the question “When you were 10 years old, compared to average, would you describe yourself as” respondents were asked to rate their body size and height in relation to their peers. The body size was classified as thinner, plumper, or approximately average (Data-Field 1687), while height was classified as shorter, taller, or approximately average (Data-Field 1697). Respondents who selected answers of “Do not know” or “Prefer not to answer” were classified as missing data. For the purposes of this study, we finally defined the following adverse early life factors that related to TL in their middle and later life: maternal smoking, lack of breastfeeding as an infant, birth weight of <2 500 g, and having a smaller body size or shorter height than peers at age 10.

### Covariates

The covariates including age (Data-Field 21022), sex (Data-Field 31), ethnicity (Data-Field 21000), level of education (Data-Field 6138), Townsend deprivation index (TDI) (Data-Field 189), body mass index (BMI) (Data-Field 21001), smoking status (Data-Field 20116), alcohol drinking status (Data-Field 20117), and white blood cell (leukocyte) count (Data-Field 30000) were incorporated into the analysis.

### Statistical Analyses

The continuous variables of baseline characteristics for the participants were presented as means ± standard deviation and compared using student *t* test, and the categorical variables were summarized as frequency and percentage and compared using chi-square test. Linear regression analysis with regression coefficients (β) and 95% confidence interval (CI) was performed to estimate the associations between frequency and types of adverse early life factors and TL. In order to investigate the correlation between the number of adverse early life factors and TL in middle and late life, participants were classified into 4 groups according to the number of aforementioned adverse early life factors (0, 1, 2, and >3) they experienced. Subsequent models were developed to account for potential confounding variables according to several relevant literature (35,37). Model 1 incorporated covariates including age (<50, 50 to <60, and ≥60 years), sex (male and female), ethnicity (White, South Asian, Black, Chinese, mixed, and any other), and BMI (<25, 25 to <30, and ≥30). Model 2 expanded the scope of Model 1 by additionally adjusting for covariates including TDI, education, categorized as college or university degree, professional qualifications, Advanced (A)-levels/Advanced Subsidiary (AS) levels/National Vocational Qualification (NVQ)/Higher National Diploma (HND)/Higher National Certificate (HNC), General Certificate of Secondary Education (GCSEs)/Ordinary (O) levels, Certificate of Secondary Education (CSEs), and none of the above; and leukocyte count (10^9^ cells per L). TDI is a census-based index of material deprivation that derived from 4 indicators including unemployment, home ownership, car ownership, and overcrowding (38), with a higher score signifying a higher level of deprivation and greater social disadvantage. Model 3 expanded the scope of Model 2 by additionally adjusting categorical variables for smoking status (Never, Previous, and Current) and alcohol drinking status (Never, Previous, and Current). In addition, when assessing the association between any individual early life risk factor (eg, maternal smoking, non-breastfed as a baby, low birthweight, thinner of comparative body size to peers at age 10, shorter of comparative height to peers at age 10) and TL, the remaining 4 adverse early life risk factors were mutually adjusted in the final model. Furthermore, stratified analyses were performed based on age, sex, BMI, and smoking status. Sensitivity analyses were performed to assess the robustness of the main findings, including using multiple imputations for missing covariates. The flexible parametric survival models with age as the time scale were employed to determine the disparity in life expectancy in individuals experiencing different number of adverse early life factors. The remaining life expectancy was estimated as the area under the predicted survival curve conditionally on survival at age 45 up to 100 years. Moreover, years of life loss and 95% CIs were calculated as the difference between the area under the 2 survival curves, between different adverse early life factors. Meanwhile, the model was adjusted for sex, ethnicity, BMI, TDI, qualification and total white blood cell count, smoking status and alcohol drinking status (39). A *p* value of < .05 was accepted statistically significant. Statistical analyses were performed using SPSS version 26.0, GraphPad Prism version 9.5 software, and Stata 17 MP.

## Results

### Population Characteristics

**Figure 1.**
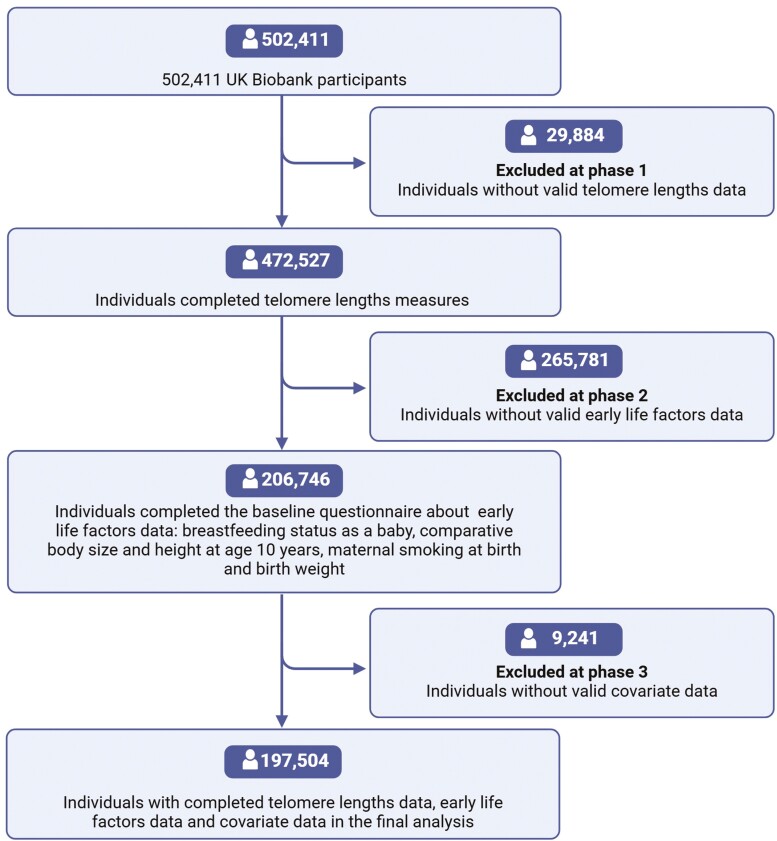
Flowchart of study participants.

As shown in Figure 1, 197 504 individuals were finally included in the analysis after excluding participants lacking valid data on TL (*n* = 29 884), early life factors (*n* = 265 781), or covariates (*n* = 9 241). The baseline characteristics of included participants were shown in Table 1, which shows a mean age of 54.8 ± 8.1 years, with 61.8% of individuals being female and 97% White. Among the total population in analysis, 28.5% of participants were exposed to maternal smoking, 29.9% had not been breastfed during infancy, 9.3% had a birth weight less than 2 500 g, 32.8% had a smaller body size than their peers at age 10, and 20.7% were shorter than their peers at age 10 (Table 1). In the univariable analysis, the following variables had a significant association with TL (Table 1), including age, sex, ethnicity, education, TDI, BMI, leukocyte count, smoking status, and alcohol drinking status; and the adverse early life factors including maternal smoking, breastfeeding, birth weight, and comparative height at age 10. In addition, only 28.1% of participants did not have any adverse early life factors, whereas 36.9%, 23.5%, and 11.5% had 1, 2, or more than 3 adverse factors, respectively (Supplementary Table 1), and the shortest TL was observed in individuals who exposed to more than 3 adverse factors.

**Table 1.** Characteristics of Participants With Leukocyte Telomere Length Measurements at Baseline

Variable	*n* (%)	Mean ± *SD*	Telomere Length (*z*-Score), *x* ± *SD*	*p* Value
Total	197 504		0.048 ± 0.997	
Age, years		54.8 ± 8.1		<.0001
<50	59 480 (30.1)		0.271 ± 0.982	
50–59	70 585 (35.7)		0.065 ± 0.978	
≥60	67 439 (34.2)		−0.166 ± 0.984	
Sex				<.0001
Male	75 392 (38.2)		−0.575 ± 0.994	
Female	122 112 (61.8)		0.113 ± 0.993	
Ethnicity				<.0001
White	191 482 (97.0)		0.039 ± 0.994	
South Asian	1 881 (1.0)		0.147 ± 1.024	
Black	1 554 (0.8)		0.596 ± 1.05	
Chinese	441 (0.2)		0.511 ± 0.972	
Mixed	1 062 (0.5)		0.243 ± 0.988	
Any other	1 084 (0.5)		0.266 ± 1.028	
Education				<.0001
College or university degree	71 181 (36.0)		0.125 ± 0.997	
Professional qualifications	9 737 (4.9)		−0.019 ± 0.988	
A-levels/AS levels/NVQ/HND/HNC	36 415 (18.4)		0.050 ± 0.988	
GCSEs/O-levels	44 249 (22.4)		0.036 ± 0.996	
CSEs	11 483 (5.8)		0.085 ± 0.977	
None of the above	24 439 (12.4)		−0.147 ± 0.993	
Townsend deprivation index		−1.516 ± 2.958		.024
Q1 (lowest)		−4.575 ± 0.616	0.044 ± 0.991	
Q2		−3.016 ± 0.412	0.039 ± 0.990	
Q3		−1.212 ± 0.708	0.054 ± 0.995	
Q4 (highest)		2.781 ± 1.970	0.055 ± 1.011	
Body mass index				<.0001
<25	70 170 (35.5)		0.118 ± 1.002	
25 to <30	80 956 (41.0)		0.026 ± 0.991	
≥30	46 378 (23.5)		−0.200 ± 0.991	
Total white blood cell (Leukocyte) count (10^9^ cells/liter)		6.839 ± 1.895		<.0001
Q1 (lowest)		4.867 ± 0.586	0.119 ± 0.990	
Q2		6.124 ± 0.286	0.058 ± 0.990	
Q3		7.182 ± 0.342	0.021 ± 0.992	
Q4 (highest)		9.227 ± 1.910	−0.007 ± 1.011	
Smoking status				<.0001
Never	113 560 (57.5)		0.085 ± 0.991	
Previous	64 898 (32.9)		−0.003 ± 0.997	
Current	19 046 (9.6)		0.002 ± 1.015	
Alcohol drinking status				.008
Never	7 158 (3.6)		0.062 ± 0.997	
Previous	6 393 (3.2)		0.012 ± 1.022	
Current	183 953 (93.1)		0.049 ± 0.996	
Maternal smoking at birth				<.0001
No	141 238 (71.5)		0.051 ± 0.996	
Yes	56 266 (28.5)		0.015 ± 1.005	
Breastfed as baby				<.0001
Yes	138 451 (70.1)		0.036 ± 0.997	
No	59 053 (29.9)		0.076 ± 0.995	
Birth weight				<.0001
Normal or high birth weight (≥2 500 g)	179 102 (90.7)		0.065 ± 0.998	
Low birth weight (<2 500 g)	18 402 (9.3)		0.006 ± 0.991	
Comparative body size to peers at age 10				.477
About average or plumper	132 755 (67.2)		0.049 ± 0.994	
Thinner	64 749 (32.8)		0.046 ± 1.001	
Comparative height to peers at age 10				.041
About average or taller	156 631 (79.3)		0.050 ± 0.996	
Shorter	40 873 (20.7)		0.039 ± 0.997	

*Notes*: A = Advanced; AS = Advanced Subsidiary; CSEs = Certificate of Secondary Education; GCSEs = General Certificate of Secondary Education; O = Ordinary; HNC = Higher National Certificate; HND = Higher National Diploma; NVQ = National Vocational Qualification; *SD* = standard deviation.

### Association of Adverse Early Life Factors With Telomere Length

**Figure 2.**
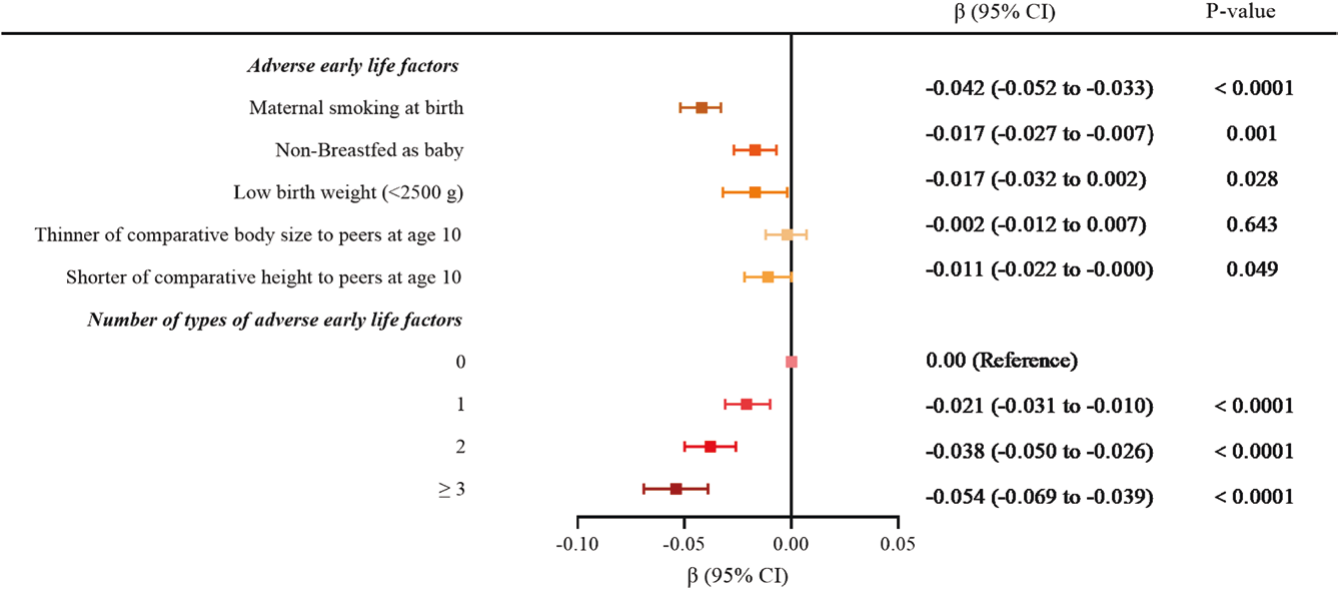
Association between early life factors and telomere length in adulthood. Adjusted for age, sex, ethnicity, body mass index, Townsend deprivation index, educational attainment, white blood cell (leukocyte) count, smoking status, and alcohol drinking status. When assessing the association between any individual early life risk factor (eg, maternal smoking, non-breastfed as a baby, low birthweight, thinner of comparative body size to peers at age 10, shorter of comparative height to peers at age 10) and TL, the remaining 4 adverse early life risk factors were mutually adjusted in the model. CI = confidence interval; TL = telomere length.

Table 2 displayed results from the minimally and fully adjusted linear regression models. Among the 5 adverse early life factors, individuals with maternal smoking exposure had the greatest association with shorter TL in the fully adjusted model (β = − 0.042; 95% CI = −0.052 to −0.033; *p* < .0001). Compared to those who were breastfed during infancy, non-breastfed participants exhibited a shorter TL (β = −0.017; 95% CI = −0.027 to −0.007; *p *= .001). Individuals who were born weighing less than 2 500 g had a shorter TL in comparison to those with average birth weight or more than 2 500 g (β = −0.017; 95% CI = −0.032 to −0.002; *p *= .028). Moreover, participants who reported shorter heights than their peers at age 10 had shorter TL compared to those who reported average or taller heights (β = − 0.011; 95% CI = −0.012 to − 0.000; *p* = .049). In regard to the relationship between body size compared to peers at age 10 and TL in adulthood, an association was observed between thinner than their peers at age 10 and shorter TL in the minimally adjusted Model 1 (β = −0.007; 95% CI = −0.020 to −0.001; *p* = .030), while such association was diminished after fully adjusting for potential confounding factors. In addition, it was demonstrated that there was an inverse correlation between the number of adverse early life factors and much shorter TL in both the minimally and fully adjusted linear regression models (all *p <* .0001). The strongest correlation was observed between shorter TL and individuals who experienced more than 3 adverse early life factors (β = − 0.054; 95% CI = −0.069 to −0.039; *p* < .0001), followed by those who experienced 2 factors (β = −0.038; 95% CI = −0.050 to 0.026; *p* < .0001), and finally, individuals who experienced only one factor (β = −0.021; 95% CI = −0.031 to 0.010; *p* < .0001) (Table 1 and Figure 2).

**Table 2.** Association of Early Life Factors and Telomere Length in Multivariable Linear Regression

Group	Model 1^*^		Model 2^†^		Model 3^‡^	
	β (95% CI)	*p* Value	β (95% CI)	*p* Value	β (95% CI)	*p* Value
Types of adverse early life factors						
Maternal smoking at birth	−0.050 (−0.063 to −0.044)	<.0001	−0.043 (−0.055 to −0.036)	<.0001	−0.042 (−0.052 to −0.033)	<.0001
Non-Breastfed as baby	−0.023 (−0.039 to −0.020)	<.0001	−0.016 (−0.031 to −0.011)	<.0001	−0.017 (−0.027 to −0.007)	.001
Low birth weight (<2 500 g)	−0.022 (−0.047 to −0.018)	<.0001	−0.016 (−0.038 to −0.008)	.002	−0.017 (−0.032 to −0.002)	.028
Thinner of comparative body size to peers at age 10	−0.007 (−0.020 to −0.001)	.030	−0.002 (−0.014 to 0.005)	.348	−0.002 (−0.012 to 0.007)	.643
Shorter of comparative height to peers at age 10	−0.013 (−0.029 to −0.008)	.001	−0.010 (−0.024 to −0.003)	.012	−0.011 (−0.022 to −0.000)	.049
Number of types of adverse early life factors						
0	Reference		Reference		Reference	
1	−0.026 (−0.037 to −0.016)	<.0001	−0.021 (−0.031 to −0.010)	<.0001	−0.021 (−0.031 to −0.010)	<.0001
2	−0.049 (−0.061 to −0.037)	<.0001	−0.038 (−0.050 to −0.026)	<.0001	−0.038 (−0.050 to −0.026)	<.0001
≥3	−0.072 (−0.087 to −0.057)	<.0001	−0.053 (−0.069 to −0.038)	<.0001	−0.054 (−0.069 to −0.039)	<.0001

*Notes*: CI = confidence interval; TL = telomere length.

^*^Covariables in model 1: age, sex, ethnicity, and body mass index.

^†^Covariables in model 2: Model 1+ Townsend deprivation index, qualification, and total white blood cell count.

^‡^Covariables in model 3: Model 2+ smoking status and alcohol drinking status; when assessing the association between any individual early life risk factor (eg, maternal smoking, non-breastfed as a baby, low birthweight, thinner of comparative body size to peers at age 10, and shorter of comparative height to peers at age 10) and TL, the remaining 4 adverse early life risk factors were mutually adjusted in the model.

### Subgroup and Sensitivity Analyses

Subgroup analyses were performed based on age, sex, BMI, and smoking status (Supplementary Tables 2–5). A notable association between maternal smoking and shorter TL was observed in the majority of subgroup analyses. Additionally, non-breastfed during infancy was significantly correlated with shorter TL in the subgroup with baseline age younger than 60 years old but not in the subgroup order than 60 years old. A negative linear correlation was observed in all subgroup analyses between the number of adverse early life factors and TL. Apart from a subgroup in current smokers, the correlation coefficient exhibited an incremental increase in absolute value as the number of adverse early life factors increased, ranging from 1 to ≥3, in comparison to individuals without any such factors. In addition, the majority of individual and combined factors were not identified to be significantly associated with TL in specific subgroup analysis such as current smokers and those participants with BMI greater than 30. Finally, sensitivity analyses after multiple imputations for critical missing data remained similar to the initial results (Supplementary Table 6), indicating the reliability of the finding.

### Life Expectancy

**Figure 3.**
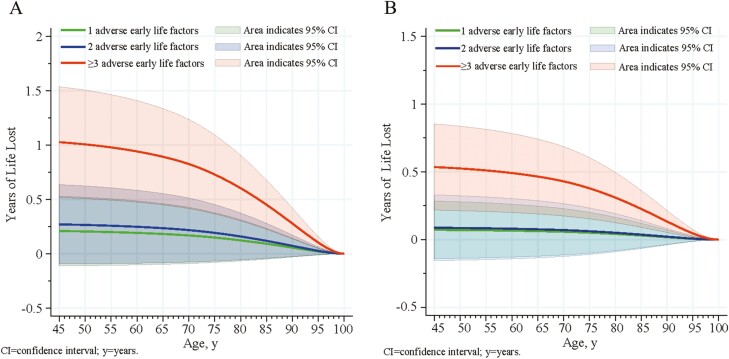
Modeling years of life lost associated with number of adverse early life factors (1, 2, ≥3) when compared to individuals with no adverse early life factors. A, before adjustment; B, after adjustment by sex, ethnicity, body mass index, Townsend deprivation index, qualification and total white blood cell count, smoking status, and alcohol drinking status.

During a median follow-up of 14.0 years, 11 226 deaths were documented. The results showed that individuals with more than 3 adverse early life factors had the greatest life expectancy loss compared to those experienced 0, 1, or 2 factors in the crude (Figure 3A) and adjusted models (Figure 3B) albeit the association was substantially attenuated in the fully adjusted model. Individuals with more than 3 adverse early life factors had a lower life expectancy than those who did not experience any factors, with a mean difference of 0.54 years (95% CI = 0.21–0.85 years) at the age of 45 and 0.49 years (95% CI = 0.20–0.78 years) at the age of 60 years (Supplementary Table 7). And specifically, participants aged 45 years who had not been exposed to any adverse early life had a remaining life expectancy of 46.40 years; however, this value decreased to 45.66 years among those individuals with the same age and exposed to at least 3 adverse early life factors (Supplementary Table 8).

## Discussion

In this large population-based observational study, we identified adverse early life factors including maternal smoking exposure, non-breastfed during infancy, birth weight less than 2 500 g, and shorter height compared to peers at age 10, were inversely correlated with TL in middle and late life. Furthermore, individuals who experienced a greater number of these factors were associated with a more likelihood of shorter TL in middle and late life, as well as a shorter life expectancy.

Previous studies have documented that an extended duration of intrauterine tobacco exposure was linked to a reduction in TL in children with a negatively linear dose–response relationship (21,22,40,41). Furthermore, this effect may persist into maturity (21,22). Limited research has been conducted on the effects of maternal smoking exposure on TL in midlife or beyond, with the majority of studies focusing on the correlation between maternal-fetal tobacco exposure and TL during infancy or childhood. Two recent studies demonstrated that maternal smoking was associated with accelerated aging phenotypes including TL (28), the Klemera–Doubal biological age and the PhenoAge algorithms in middle and later life (42), which also supported our findings on such relationship. Tobacco byproducts transported across the placenta by maternal smoker have the potential to affect fetal development physilogically (43,44) induce oxidative stress and inflammation, and ultimately may shorten TL (45).

The majority of previous studies mainly focused on the correlation between breastfeeding during infancy and TL among childhood and adolescence, but not in adulthood. One observational study with relatively small sample size failed to establish a correlation between the duration of breastfeeding and the TL in adults (15). Our observational study of 197 504 UK Biobank participants revealed that those who were not breastfed during infancy had substantially shorter TL in middle age and older adulthood than those who were breastfed during infancy. This phenomenon could potentially be explained by the protective impact of lactation on the development of the infant microbiota and immunity (46). Additionally, breastfeeding may affect the development of nutritious dietary habits during the early stages of life, potentially with a lasting effect (47). Nevertheless, such an association between breastfeeding and TL was not statistically significant in the subgroup of participants aged 60 years or older at the recruitment. The possible explanations for the observed phenomenon were that the proportion of individuals who had experienced breastfeeding was greater among the older generation than among the younger generation (48). However, the disparity was not substantial enough to warrant statistical significance regarding the proportions of breastfed populations in the 2 groups. Conversely, increased rates of breastfeeding among the older adults could potentially be interpreted as a reflection of a lower socioeconomic status, which has been linked to reduced durations of breastfeeding, as well as shortened TL for cord blood and placenta (49).

In previous studies, the relationship between birth weight and TL in adults was far from consistent. In a comparative study involving 120 healthy male participants aged 21 to 25 years old, specifically including 55 individuals with low birth weight and 65 individuals with normal birth weight, no significant difference was observed in TL between these 2 groups (50). An additional investigation encompassing 3 small cohorts with over 2 000 individuals of diverse age groups also yielded inconclusive results regarding TL in adulthood between normal controls and those with low birth weight (31). In contrast, a correlation was identified between elevated birth weight and long TLs in early adulthood (around 21 years of age) among 1 562 Filipinos in a cohort study (29). In addition, 179 individuals with extremely low birth weight (< 1 000 g) had a shorter TL in adulthood than 145 control participants with normal birth weight (51), according to another study. Our investigation revealed that participants who were born weighing less than 2 500 g had shorter TL in middle and elderly age when compared to those who were born weighing average or more than 2 500 g. However, such association was primarily observed among male individuals, never smokers, those who were younger than 50 at the recruitment, and had a BMI lower than 30. The reported short cord lengths in older individuals (52), obese individuals (53), and smokers (54), are plausibly responsible for the inconsistent results observed in the subgroup analysis. In addition to early life risk factors, TL could be influenced by multifaceted factors, thus such mild relationship between early life factor and TL in adulthood may be progressively obscured due to environmental and lifestyle modifications.

The body size and height during childhood and adolescence have been rarely linked to TL in adulthood with consistent conclusion in previous studies. A birth cohort study with 832 individuals aged 49 to 51 years old showed a positive correlation between the height at the age of 9 and their TL in adulthood among females but not males (30). In contrast, an additional cross-sectional investigation encompassing 330 boys and 393 girls aged 5 to 12 years showed a positive correlation between the boys’ height, but not girls’, and TL at their current age (55). Based on our findings, it appeared that individuals with a shorter stature at the age of 10 exhibited a shorter TL during middle and late adulthood compared to those with average or greater height at that time. This finding suggested that being shorter than one’s peers in childhood and adolescence could potentially be an unfavorable early life factor that was linked to shorter TL in middle and late life. Similarly, individuals who were relatively thinner than their peers at age 10 may experience certain disadvantages in early life that were related to TL in middle to late life, in contrast to those who were plumper or of average weight albeit such relationship was even diminished after controlling for potential confounding factors. A study comprising 406 healthy Chinese children aged 6 to 11 years revealed a positive correlation between skeletal muscle mass and bone mass with leukocyte TL (56). This finding may provide support for our own that being thinner than peers at the age of 10 was a detrimental factor associated with a shorter TL in middle age and later years. Nevertheless, cross-sectional studies and meta-analyses have indicated a potential correlation between obesity and reduced TL in children and adolescents (26,57,58). Regarding the relationship between height and size in adolescence and TL in middle and late life, there is currently no consensus; therefore, additional extensive and high-quality studies would be warranted in the future.

Given the fact that genomic instability is widely recognized as distinctive indicator of aging (59), and extensive evidence from epidemiological studies has linked shortened TL to multiple aging-related diseases and mortality (1–3), thus motivating us to ponder the relationship between early life factors and life expectancy in middle and late life. Previous studies have established a robust link between life expectancy and genetic predisposition, social environments, and lifestyle behaviors among population, such as gender (60), smoking (61), ethnicity (62) and economic status (63), etc. Certain detrimental factors mentioned above possess the capacity to reduce life expectancy by a number of years, or even a decade. According to our findings, these early life factors might exert an enduring influence on life expectancy in old age. Specifically, when considering all confounding variables, it was possible that individuals who were exposed to 3 or more adverse early life factors would have a 0.54-year shorter life expectancy at age 45 years, in comparison to those who were not exposed to any such factors in their early lives. Although the effect size of the lost years of life may be modest in magnitude, the potential consequences for global population health and the aging process among the global population could be amplified. In addition to our findings, a number of recent studies have discovered correlations between certain early risk factors and the development of age-related illnesses and mortality (28,42,64). Collectively, there is some evidence, derived from our research and that of other studies, that interventions targeting early life risk factors may contribute to the improvement of healthier populations and a longer life expectancy. Nevertheless, further studies would be warranted to validate the correlation between adverse early life factors and aging process, life expectancy and mortality, in addition to identify any potential mechanisms embedded in these associations.

## Strengths and Limitations

Majority of previous studies that primarily examined the relationship between individual early life factors and TL in childhood or adolescence, this investigation aimed to observe the relationship between 5 individual or combined early life factors and TL during middle and late life, in addition to life expectancy. Nevertheless, certain limitations should be acknowledged. To start with, the UK Biobank cohort predominantly consisted of White participants (approximately 97%), thus restricting the interpretation of the findings to other ethnic backgrounds. Furthermore, all information regarding early life factors—such as breastfeeding, maternal smoking exposure, and birth weight—was self-reported, thus introducing potential recall bias that can be attributed to the subject or others. Individual perceptual bias was observed in the comparison of height and weight at age 10 years to that of peers. Additionally, residual confounding variables, such as genetic, behavioral, and environmental influences on TL (11,12,35,65,66), may not have been considered in the study. In addition, survival bias may be inevitable as participants with more adverse life factors exposure may have experienced more probability of premature mortality. Although our epidemiological investigations have established a correlation between detrimental early life factors and shorter TL and reduced life expectancy in middle and late life, more comprehensive examination of the underlying genetic or molecular mechanisms deserve further exploration.

## Conclusion

The study demonstrated that early adverse life factors, including maternal smoking exposure, non-breastfed during infancy, low birth weight, and shorter than peers at age 10, were associated with shorter TL and life expectancy in middle and late life. TL and life expectancy in middle and late life are also negatively correlated with the quantity of adverse early life factors. Therefore, imparting knowledge to families regarding the significance of preventing early exposure to detrimental life circumstances—for instance, advocating for breastfeeding, educating gestational women against smoking, and enhancing nutritional intake during fetus, infants, childhood and adolescents—can contribute to better long-term health outcomes for the global population health.

## Supplementary Material

igae070_suppl_Supplementary_Material

## Data Availability

Data from the UK Biobank cannot be shared publicly, however, data are available from the UK Biobank Institutional Data Access/Ethics Committee (contact via http://www.ukbiobank.ac.uk/or contact by email at ku.ca.knaboibku@ssecca) for researchers who meet the criteria for access to confidential data.
