# Supplementary material for: Association Between Adverse Early Life Factors and Telomere Length in Middle and Late Life
Source: Innov Aging. 2024 Aug 10;8(9):igae070. doi: 10.1093/geroni/igae070 (PMC11441326; doi:10.1093/geroni/igae070)
Supplement: igae070_suppl_Supplementary_Material [file igae070_suppl_supplementary_material.docx]

***Innovation in Aging* Supplementary Material: Association Between Adverse Early Life Factors and Telomere Length in Middle and Late Life.**

Supplementary Table 1. Participant demographics partitioned by number of types of adverse early life factors.

| **Variable** | **Number of types of adverse early-life factors** | | | | |
| --- | --- | --- | --- | --- | --- |
|  | **0**  **(n=55558, 28.1%)** | **1**  **(n=72839, 36.9%)** | **2**  **(n=46419, 23.5%)** | **≥3**  **(n=22688, 11.5%)** | ***p-value*** |
| **Log (T/S ratio), z-score, mean ± SD** | 0.06±1.0 | 0.05±1.0 | 0.04±1.0 | 0.03±1.0 | 0.004 |
| **Age, n (%)** |  |  |  |  | <0.0001 |
| <50 | 14428 (26.0) | 21759 (29.9) | 15398 (33.2) | 7895 (34.8) |  |
| 50–59 | 19927 (35.9) | 26000 (35.7) | 16512 (35.6) | 8146 (35.9) |  |
| ≥ 60 | 21203 (38.2) | 25080 (34.4) | 14509 (31.3) | 6647 (29.3) |  |
| **Sex, n (%)** |  |  |  |  | <0.0001 |
| Male | 21650 (39.0) | 28303 (38.9) | 17469 (37.6) | 7970 (35.1) |  |
| Female | 33908 (61.0) | 44536 (61.1) | 28950 (62.4) | 14718 (64.9) |  |
| **Ethnicity, n (%)** |  |  |  |  | <0.0001 |
| White | 53262 (95.9) | 70676 (97.0) | 45287 (97.6) | 22257 (98.1) |  |
| South Asian | 733 (1.3) | 699 (1.0) | 346 (0.7) | 103 (0.5) |  |
| Black | 724 (1.3) | 553 (0.8) | 227 (0.5) | 50 (0.2) |  |
| Chinese | 158 (0.3) | 161 (0.2) | 89 (0.2) | 33 (0.1) |  |
| Mixed | 267 (0.5) | 361 (0.5) | 272 (0.6) | 162 (0.7) |  |
| Any other | 414 (0.7) | 389 (0.5) | 198 (0.4) | 83 (0.4) |  |
| **Education, n (%)** |  |  |  |  | <0.0001 |
| College or University degree | 22198 (40.0) | 26684 (36.6) | 15602 (33.6) | 6697 (29.5) |  |
| Professional Qualifications | 2869 (5.2) | 3573 (4.9) | 2214 (4.8) | 1081 (4.8) |  |
| A-levels/AS levels/NVQ/HND/HNC | 10021 (18.0) | 13483 (18.5) | 8699 (18.7) | 4212 (18.6) |  |
| GCSEs/O-levels | 12221 (22.0) | 16350 (22.4) | 10432 (22.5) | 5246 (23.1) |  |
| CSEs | 2585 (4.7) | 4043 (5.6) | 3148 (6.8) | 1707 (7.5) |  |
| None of the above | 5664 (10.2) | 8706 (12.0) | 6324 (13.6) | 3745 (16.5) |  |
| **Townsend deprivation, n (%)** |  |  |  |  | <0.0001 |
| Q1 (lowest) | 14628 (26.3) | 18583 (25.5) | 11229 (24.2) | 5145 (22.7) |  |
| Q2 | 14335 (25.8) | 18302 (25.1) | 11528 (24.8) | 5283 (23.3) |  |
| Q3 | 13936 (25.1) | 18280 (25.1) | 11542 (24.9) | 5664 (25.0) |  |
| Q4 (highest) | 12659 (22.8) | 17674 (24.3) | 12120 (26.1) | 6596 (29.1) |  |
| **Body mass index, n (%)** |  |  |  |  | <0.0001 |
| <25 | 18327 (33.0) | 25925 (35.6) | 17213 (37.1) | 8705 (38.4) |  |
| 25 to <30 | 23522 (42.3) | 29783 (40.9) | 18616 (40.1) | 9035 (39.8) |  |
| ≥ 30 | 13709 (24.7) | 17131 (23.5) | 10590 (22.8) | 4948 (21.8) |  |
| **Smoking status, n (%)** |  |  |  |  | <0.0001 |
| Never | 31724 (57.1) | 41598 (57.1) | 26922 (58.0) | 13316 (58.7) |  |
| Previous | 18867 (34.0) | 24230 (33.3) | 14876 (32.0) | 6925 (30.5) |  |
| Current | 4967 (8.9) | 7011 (9.6) | 4621 (10.0) | 2447 (10.8) |  |
| **Alcohol drinking status, n (%)** |  |  |  |  | <0.0001 |
| Never | 2220 (4.0) | 2530 (3.5) | 1613 (3.5) | 795 (3.5) |  |
| Previous | 1559 (2.8) | 2330 (3.2) | 1610 (3.5) | 894 (3.9) |  |
| Current | 51779 (93.2) | 67979 (93.3) | 43196 (93.1) | 20999 (92.6) |  |
| **Total white blood cell (Leukocyte) count (10^9 cells/Litre), mean ± SD** | 6.7±1.8 | 6.8±1.9 | 6.9±1.9 | 7.0±1.9 | <0.0001 |
| **Maternal smoking at birth, n (%)** |  |  |  |  | <0.0001 |
| No | 55558 (100.0) | 54514 (74.8) | 24394 (52.6) | 6772 (29.8) |  |
| Yes | 0 (0.0) | 18325 (25.2) | 22025 (47.4) | 15916 (70.2) |  |
| **Breastfed as baby, n (%)** |  |  |  |  | <0.0001 |
| Yes | 55558 (100.0) | 53607 (73.6) | 23104 (49.8) | 6182 (27.2) |  |
| No | 0 (0.0) | 19232 (26.4) | 23315 (50.2) | 16506 (72.8) |  |
| **Birth weight, n (%)** |  |  |  |  | <0.0001 |
| Normal or high birth weight (≥ 2500 g) | 55558 (100.0) | 69646 (95.6) | 40251 (86.7) | 11991 (65.5) |  |
| Low birth weight (<2500 g) | 0 (0.0) | 3193 (4.4) | 6168 (13.3) | 9041 (39.8) |  |
| **Comparative body size to peers at age 10, n (%)** |  |  |  |  | <0.0001 |
| About average or plumper | 55558 (100.0) | 50577 (69.4) | 21411 (46.1) | 5209 (23.0) |  |
| Thinner | 0 (0.0) | 22262 (30.6) | 25008 (53.9) | 17479 (77.0) |  |
| **Comparative height to peers at age 10, n (%)** |  |  |  |  | <0.0001 |
| About average or taller | 55558 (100.0) | 63012 (86.5) | 30097 (64.8) | 7964 (35.1) |  |
| Shorter | 0 (0.0) | 9827 (13.5) | 16322 (35.2) | 14724 (64.9) |  |

Abbreviations: A, Advanced; AS, Advanced Subsidiary; CSEs, Certificate of Secondary Education; GCSEs, General Certificate of Secondary Education; O, Ordinary; HNC, Higher National Certificate; HND, Higher National Diploma; NVQ, National Vocational Qualification; SD, standard deviation.

Supplementary Table 2. Association of early life factors and telomere length in multivariable linear regression by age.

| Group | Age<50 | | 50≤Age＜60 | | Age≥60 | |
| --- | --- | --- | --- | --- | --- | --- |
|  | Multi-adjusted β (95% CI) | *p-value* | Multi-adjusted β (95% CI) | *p-value* | Multi-adjusted β (95% CI) | *p-value* |
| **Types of adverse early life factors** |  |  |  |  |  |  |
| Maternal smoking at birth | -0.064 (-0.082 to -0.047) | ＜0.0001 | -0.033 (-0.049 to -0.018) | ＜0.0001 | -0.032 (-0.048 to -0.015) | ＜0.0001 |
| Non-breastfed as baby | -0.017 (-0.033 to -0.001） | 0.041 | -0.023 (-0.040 to -0.007） | 0.005 | -0.008 (-0.026 to 0.010） | 0.397 |
| Low birth weight (<2500 g) | -0.030 (-0.060 to -0.000) | 0.049 | -0.014 (-0.039 to 0.011) | 0.275 | -0.015 (-0.039 to 0.010) | 0.237 |
| Thinner of comparative body size to peers at age 10 | 0.009 (-0.008 to 0.026) | 0.283 | -0.007 (-0.022 to 0.009) | 0.399 | -0.008 (-0.024 to 0.008) | 0.301 |
| Shorter of comparative height to peers at age 10 | -0.001 (-0.020 to 0.019) | 0.945 | -0.018 (-0.036 to 0.000) | 0.052 | -0.013 (-0.032 to 0.005) | 0.164 |
| **Number of types of adverse early life factors** |  |  |  |  |  |  |
| 0 | Reference |  | Reference |  | Reference |  |
| 1 | -0.009 (-0.030 to 0.011) | 0.381 | -0.032 (-0.050 to -0.014) | ＜0.0001 | -0.019 (-0.037 to -0.001) | 0.035 |
| 2 | -0.040 (-0.062 to -0.018) | ＜0.0001 | -0.046 (-0.066 to -0.026) | ＜0.0001 | -0.028 (-0.049 to -0.007) | 0.008 |
| ≥3 | -0.057 (-0.084 to -0.030) | ＜0.0001 | -0.058 (-0.083 to -0.033) | ＜0.0001 | -0.049 (-0.076 to -0.022) | ＜0.0001 |

Abbreviations: CI, confidence interval.

Covariables in model: sex, ethnicity, body mass index, Townsend deprivation index, qualification, total white blood cell count, smoking status and alcohol drinking status. When assessing the association between any individual early life risk factor (e.g., maternal smoking, non-breastfed as a baby, low birthweight, thinner of comparative body size to peers at age 10, shorter of comparative height to peers at age 10) and TL, the remaining four adverse early life risk factors were mutually adjusted in the model.

Supplementary Table 3. Association of early life factors and telomere length in multivariable linear regression by sex.

| Group | Male | |  | Female | |
| --- | --- | --- | --- | --- | --- |
|  | Multi-adjusted β (95% CI) | *p-value* |  | Multi-adjusted β (95% CI) | *p-value* |
| **Types of adverse early life factors** |  |  |  |  |  |
| Maternal smoking at birth | -0.032 (-0.047 to -0.016) | ＜0.0001 |  | -0.049 (-0.061 to -0.036) | ＜0.0001 |
| Non-Breastfed as baby | -0.007 (-0.023 to 0.009） | 0.408 |  | -0.023 (-0.036 to -0.011） | ＜0.0001 |
| Low birth weight (<2500 g) | -0.034 (-0.061 to -0.006) | 0.017 |  | -0.011 (-0.029 to 0.007) | 0.237 |
| Thinner of comparative body size to peers at age 10 | -0.006 (-0.021 to 0.009) | 0.438 |  | -0.001 (-0.013 to 0.011) | 0.910 |
| Shorter of comparative height to peers at age 10 | 0.000 (-0.017 to 0.018) | 0.970 |  | -0.017 (-0.031 to 0.004) | 0.012 |
| **Number of types of adverse early life factors** |  |  |  |  |  |
| 0 | Reference |  |  | Reference |  |
| 1 | -0.020 (-0.037 to 0.002) | 0.026 |  | -0.022 (-0.036 to -0.009) | 0.002 |
| 2 | -0.027 (-0.047 to -0.008) | 0.006 |  | -0.046 (-0.062 to -0.031) | ＜0.0001 |
| ≥3 | -0.040 (-0.065 to -0.015) | 0.002 |  | -0.064 (-0.083 to -0.045) | ＜0.0001 |

Abbreviations: CI, confidence interval. Covariables in model: age, ethnicity, body mass index, Townsend deprivation index, qualification, total white blood cell count, smoking status and alcohol drinking status. When assessing the association between any individual early life risk factor (e.g., maternal smoking, non-breastfed as a baby, low birthweight, thinner of comparative body size to peers at age 10, shorter of comparative height to peers at age 10) and TL, the remaining four adverse early life risk factors were mutually adjusted in the model.

Supplementary Table 4. Association of early life factors and telomere length in multivariable linear regression by body mass index.

| Group | BMI<25 | |  | 25≤BMI＜30 | |  | ≥ 30 | |
| --- | --- | --- | --- | --- | --- | --- | --- | --- |
|  | Multi-adjusted β (95% CI) | *p-value* |  | Multi-adjusted β (95% CI) | *p-value* |  | Multi-adjusted β (95% CI) | *p-value* |
| **Types of adverse early life factors** |  |  |  |  |  |  |  |  |
| Maternal smoking at birth | -0.043 (-0.060 to -0.026) | ＜0.0001 |  | -0.036 (-0.051 to -0.021) | ＜0.0001 |  | -0.051 (-0.070 to -0.032) | ＜0.0001 |
| Non-Breastfed as baby | -0.008 (-0.025 to 0.008） | 0.304 |  | -0.019 (-0.035 to -0.004） | 0.012 |  | -0.026 (-0.046 to -0.006） | 0.010 |
| Low birth weight (<2500 g) | -0.026 (-0.052 to 0.000) | 0.047 |  | -0.026 (-0.050 to -0.002) | 0.031 |  | 0.011 (-0.193 to 0.040) | 0.480 |
| Thinner of comparative body size to peers at age 10 | 0.002 (-0.013 to 0.017) | 0.817 |  | -0.001 (-0.016 to 0.013) | 0.855 |  | -0.011 (-0.032 to 0.010) | 0.300 |
| Shorter of comparative height to peers at age 10 | -0.001 (-0.019 to 0.017) | 0.888 |  | -0.014 (-0.031 to 0.003) | 0.100 |  | -0.021 (-0.043 to 0.002) | 0.076 |
| **Number of types of adverse early life factors** |  |  |  |  |  |  |  |  |
| 0 | Reference |  |  | Reference |  |  | Reference |  |
| 1 | -0.022 (-0.041 to -0.003) | 0.021 |  | -0.031 (-0.048 to -0.014) | ＜0.0001 |  | 0.000 (-0.022 to 0.022) | 0.998 |
| 2 | -0.029 (-0.050 to -0.009) | 0.005 |  | -0.043 (-0.061 to -0.024) | ＜0.0001 |  | -0.045 (-0.070 to -0.020) | ＜0.0001 |
| ≥3 | -0.045 (-0.070 to -0.020) | ＜0.0001 |  | -0.053 (-0.076 to -0.002) | ＜0.0001 |  | -0.073 (-0.105 to -0.041) | ＜0.0001 |

Abbreviations: CI, confidence interval. Covariables in model: age, sex, ethnicity, Townsend deprivation index, qualification, total white blood cell count, smoking status and alcohol drinking status. When assessing the association between any individual early life risk factor (e.g., maternal smoking, non-breastfed as a baby, low birthweight, thinner of comparative body size to peers at age 10, shorter of comparative height to peers at age 10) and TL, the remaining four adverse early life risk factors were mutually adjusted in the model.

Supplementary Table 5. Association of early life factors and telomere length in multivariable linear regression by smoking status.

| Group | Never | |  | Previous | |  | Current | |
| --- | --- | --- | --- | --- | --- | --- | --- | --- |
|  | Multi-adjusted β (95% CI) | *p-value* |  | Multi-adjusted β (95% CI) | *p-value* |  | Multi-adjusted β (95% CI) | *p-value* |
| **Types of adverse early life factors** |  |  |  |  |  |  |  |  |
| Maternal smoking at birth | -0.049 (-0.061 to -0.036) | ＜0.0001 |  | -0.028 (-0.045 to -0.011) | 0.001 |  | -0.052 (-0.083 to -0.022) | 0.001 |
| Non-Breastfed as baby | -0.017 (-0.029 to -0.004） | 0.009 |  | -0.015 (-0.032 to 0.002） | 0.089 |  | -0.023 (-0.054 to 0.008） | 0.141 |
| Low birth weight (<2500 g) | -0.020 (-0.039 to -0.001) | 0.041 |  | -0.017 (-0.045 to 0.010) | 0.214 |  | 0.006 (-0.043 to 0.055) | 0.814 |
| Thinner of comparative body size to peers at age 10 | 0.008 (-0.004 to 0.020) | 0.209 |  | -0.023 (-0.040 to -0.007) | 0.005 |  | 0.007 (-0.024 to 0.038) | 0.647 |
| Shorter of comparative height to peers at age 10 | -0.012 (-0.026 to 0.002) | 0.086 |  | -0.009 (-0.028 to 0.010) | 0.333 |  | -0.004 (-0.040 to 0.032) | 0.825 |
| **Number of types of adverse early life factors** |  |  |  |  |  |  |  |  |
| 0 | Reference |  |  | Reference |  |  | Reference |  |
| 1 | -0.024 (-0.038 to -0.010) | 0.001 |  | -0.015 (-0.034 to 0.003) | 0.102 |  | -0.017 (-0.054 to 0.019) | 0.348 |
| 2 | -0.034 (-0.050 to -0.018) | ＜0.0001 |  | -0.041 (-0.062 to -0.020) | ＜0.0001 |  | -0.055 (-0.095 to -0.015) | 0.008 |
| ≥3 | -0.056 (-0.076 to -0.036) | ＜0.0001 |  | -0.056 (-0.083 to -0.029) | ＜0.0001 |  | -0.037 (-0.086 to 0.012) | 0.135 |

Abbreviations: CI, confidence interval. Covariables in model: age, sex, ethnicity, body mass index, Townsend deprivation index, qualification, total white blood cell counts and alcohol drinking status. When assessing the association between any individual early life risk factor (e.g., maternal smoking, non-breastfed as a baby, low birthweight, thinner of comparative body size to peers at age 10, shorter of comparative height to peers at age 10) and TL, the remaining four adverse early life risk factors were mutually adjusted in the model.

Supplementary Table 6. Association of early life factors and telomere length in multivariable linear regression with multiple imputations.

| Group | Model 1^a^ | |  | Model 2^b^ | |  | Model 3^c^ | |
| --- | --- | --- | --- | --- | --- | --- | --- | --- |
|  | β (95% CI) | *p-value* |  | β (95% CI) | *p-value* |  | β (95% CI) | *p-value* |
| **Types of adverse early life factors** |  |  |  |  |  |  |  |  |
| Maternal smoking at birth | -0.052 (-0.062 to -0.043) | ＜0.0001 |  | -0.045 (-0.054 to -0.035) | ＜0.0001 |  | -0.044 (-0.053 to -0.035) | ＜0.0001 |
| Non-Breastfed as baby | -0.024 (-0.034 to -0.015） | ＜0.0001 |  | -0.017 (-0.027 to -0.008) | ＜0.0001 |  | -0.018 (-0.027 to -0.009) | ＜0.0001 |
| Low birth weight (<2500 g) | -0.022 (-0.036 to -0.007) | 0.004 |  | -0.016 (-0.030 to -0.001) | 0.038 |  | -0.017 (-0.031 to -0.002) | 0.025 |
| Thinner of comparative body size to peers at age 10 | -0.005 (-0.014 to 0.004) | 0.306 |  | 0.000 (-0.009 to 0.009) | 0.967 |  | -0.001 (-0.010 to 0.009) | 0.902 |
| Shorter of comparative height to peers at age 10 | -0.014 (-0.025 to -0.004) | 0.007 |  | -0.012 (-0.022 to -0.001) | 0.030 |  | -0.012 (-0.023 to -0.001) | 0.026 |
| **Number of types of adverse early life factors** |  |  |  |  |  |  |  |  |
| 0 | Reference |  |  | Reference |  |  | Reference |  |
| 1 | -0.026 (-0.037 to -0.016) | ＜0.0001 |  | -0.021 (-0.031 to -0.010) | ＜0.0001 |  | -0.021 (-0.031 to -0.010) | ＜0.0001 |
| 2 | -0.049 (-0.061 to -0.038) | ＜0.0001 |  | -0.038 (-0.050 to -0.026) | ＜0.0001 |  | -0.039 (-0.050 to -0.027) | ＜0.0001 |
| ≥3 | -0.073 (-0.088 to -0.058) | ＜0.0001 |  | -0.055 (-0.070 to -0.040) | ＜0.0001 |  | -0.056 (-0.070 to -0.041) | ＜0.0001 |

Abbreviations: CI, confidence interval.

^a^ Covariables in model 1: age, sex, ethnicity, body mass index. ^b^ Covariables in model 2: Model 1+Townsend deprivation index, qualification and total white blood cell count. ^c^ Covariables in model 3: Model 2+smoking status and alcohol drinking status; when assessing the association between any individual early life risk factor (e.g., maternal smoking, non-breastfed as a baby, low birthweight, thinner of comparative body size to peers at age 10, shorter of comparative height to peers at age 10) and TL, the remaining four adverse early life risk factors were mutually adjusted in the model.

Supplementary Table 7. Years of life lost at ages 45 and 60 years by adverse early life factors.

| Group | Crude model | | |  | Adjusted model | | |
| --- | --- | --- | --- | --- | --- | --- | --- |
|  | Years of life lost (95% CI), 45y |  | Years of life lost (95% CI), 60y |  | Years of life lost (95% CI), 45y |  | Years of life lost (95% CI), 60y |
| Number of types of adverse early life factors |  |  |  |  |  |  |  |
| 0 | Ref. |  | Ref. |  | Ref. |  | Ref. |
| 1 | 0.21 (-0.11, 0.53) |  | 0.19 (-0.10, 0.49) |  | 0.07 (-0.14, 0.28) |  | 0.07 (-0.13, 0.26) |
| 2 | 0.27 (-0.10, 0.64) |  | 0.25 (-0.09, 0.58) |  | 0.08 (-0.15, 0.33) |  | 0.08 (-0.14, 0.30) |
| ≥3 | 1.03 (0.52, 1.54) |  | 0.94 (0.47, 1.41) |  | 0.54 (0.21, 0.85) |  | 0.49 (0.20, 0.78) |

Abbreviations: CI, confidence interval.

The crude model was not adjusted. The adjusted model was adjusted for sex, ethnicity, body mass index, Townsend deprivation index, qualification and total white blood cell count, smoking status and alcohol drinking status.

Supplementary Table 8. Remaining life expectancy at ages 45 and 60 years by adverse early life factors.

| Group | Crude model | | | | Adjusted model | | | |
| --- | --- | --- | --- | --- | --- | --- | --- | --- |
|  | ERL (95% CI), 45y |  | ERL (95% CI), 60y | ERL (95% CI), 45y | |  | ERL (95% CI), 60y |  |
| Number of types of adverse early life factors |  |  |  |  | |  |  |  |
| 0 | 45.95 (45.52, 46.38) |  | 31.65 (31.24, 32.06) | 46.40 (46.00, 46.79) | |  | 32.12 (31.75, 32.50) |  |
| 1 | 45.74 (45.32, 46.16) |  | 31.45 (31.05, 31.86) | 46.30 (45.91, 46.69) | |  | 32.03 (31.66, 32.40) |  |
| 2 | 45.68 (45.22, 46.14) |  | 31.40 (31.00, 31.84) | 46.28 (45.85, 46.70) | |  | 32.01 (31.61, 32.42) |  |
| ≥3 | 45.02 (44.46, 45.58) |  | 30.79 (30.26, 31.33) | 0.54 (0.21, 0.85) | |  | 0.49 (0.20, 0.78) |  |

Abbreviations: CI, confidence interval. ERL=estimated remaining life expectancy.

The crude model was not adjusted. The adjusted model was adjusted for sex, ethnicity, body mass index, Townsend deprivation index, qualification and total white blood cell count, smoking status and alcohol drinking status.
